# Reporting and handling of missing data in published studies of co-morbid hypertension and diabetes among people living with HIV/AIDS: a systematic review

**DOI:** 10.1186/s12874-025-02630-1

**Published:** 2025-07-30

**Authors:** Peter Vanes Ebasone, Nasheeta Peer, Anastase Dzudie, Johney Melpsa, Merveille Foaleng, Andre Pascal Kengne

**Affiliations:** 1 Department of Medicine, University of Cape Town, Cape Town, South Africa; 2 Clinical Research Education, Networking and Consultancy (CRENC), Yaounde, Cameroon; 3 Non-Communicable Disease Research Unit, South African Medical Research Council, Cape Town, South Africa; 4 Faculty of Medicine and Biomedical Sciences, University of Yaounde I, Yaounde, Cameroon; 5 Lown Scholars Program, Harvard T. H. Chan School of Public Health, Boston, United States of America

**Keywords:** Missing data, Hypertension, Diabetes, Cross-sectional, HIV/AIDS

## Abstract

**Background:**

As hypertension and diabetes emerge as co-morbidities among people living with HIV/AIDS (PLWH), the need for robust epidemiological research to inform policy and action is imperative. Proper reporting and handling of missing data are crucial in such studies to avoid loss of statistical power and precision and generate unbiased results. We aimed to assess the reporting and handling of missing data in published studies of co-morbid hypertension and diabetes among PLWH.

**Methods:**

We searched in PubMed for cross-sectional studies of co-morbid hypertension and diabetes among PLWH published worldwide between January 1990 and June 2023. We extracted data on reporting of missing data (quantity, type, where it occurred, and any bias assessment) and how it was handled.

**Results:**

Of 2179 records identified, 154 studies were included among which 53 (34.4%) reported missing data, primarily within exposure variables such as CD4 count and viral load. Only 19 of these studies (37.7%) cited reasons for missingness, predominantly attributed to lack of documentation and non-response. Out of the 24 (45.5%) studies that detailed how they handled missing data, the majority (16 studies; 30.2%) used complete case analysis. Only 5/53 studies (9.43%) adopted multiple imputation methods. The potential biases introduced by missing data were acknowledged in only 12/53 (22.6%) studies.

**Conclusion:**

The reporting and handling of missing data in hypertension and diabetes studies among PLWH are currently suboptimal. Enhanced understanding of why data is missing and choosing appropriate methods to address it is paramount to reduce potential biases. Adopting and adhering to comprehensive guidelines for managing missing data is a pressing need and will ensure that more accurate results are better represented in PLWH population.

**Supplementary Information:**

The online version contains supplementary material available at 10.1186/s12874-025-02630-1.

## Background

As life expectancy increases for people living with HIV (PLWH) due to effective antiretroviral therapy (ART) [1, 2], they face a higher risk of developing cardiometabolic diseases (CMDs) such as hypertension and diabetes [3]. To tackle this growing concern, robust studies of co-morbid hypertension and diabetes among PLWH are warranted to enable both clinicians and policymakers to formulate evidence-based care strategies and health policies. However, the accuracy and validity of such data depends on employed methodologies such as the methods for handing missing information [4].

Missing data is a pervasive challenge in medical research and can significantly impact the validity and generalizability of findings, particularly in studies where critical variables such as exposures and confounders are affected [5]. Depending on the study design, research question, and the nature of the exposure-outcome relationship, missing data can influence conclusions in varying and significant ways. For example, in studies exploring relationships between HIV-related factors (e.g., CD4 count, viral load and ART) and CMDs such as hypertension and diabetes, missingness in key variables can lead to biased estimates, reduced statistical power, and compromised reliability of findings [5, 6]. These variables, CD4 count and viral load, are of particular interest in research on CMDs among PLWH, as it has been suggested that HIV and ART influence CMD through mechanisms such as chronic inflammation, immune activation, and the direct metabolic effects of ART [7]. Although causal inference may not always be the primary goal, addressing missing data is essential for ensuring the robustness of observed associations and generating meaningful insights [4, 5]. This is particularly relevant in studies that are largely questionnaire-based, where nonresponse bias and undocumented missingness are common [8]. Employing rigorous methods such as multiple imputation for Missing at Random (MAR) data can mitigate biases, preserve the integrity of statistical analyses, and enhance the overall validity of findings [4–6]. These efforts are critical for providing a strong foundation for future research and guiding evidence-based interventions.

Despite several reviews [9–11] emphasising the importance of reporting and addressing missing data, missing data remain frequently observed in medical research, but the practice of addressing this is improving slowly [12]. This is particularly pertinent in observational research [13], where there is limited regulatory framework to guide the methodology and analyses are frequently adjusted for confounders with missing values [10]. Despite the well-acknowledged fact that missing data can lead to reduced statistical power and introduce bias, the potential influence of missing data on scientific conclusions is frequently overlooked by researchers [5]. For example, unaddressed missing data in key variables may not only distort the results of statistical analyses but also lead to misinterpretation of findings, particularly when assumptions about the nature of missingness are inadequately tested [5, 6, 11]. Although many journals require authors to justify their methods for addressing missing data and to provide explanations for the extent of missingness [14], inconsistencies in adherence to these guidelines remain a significant challenge. Such gaps in transparency and reproducibility highlight the critical need for more rigorous reporting and handling of missing data to strengthen the credibility of scientific research.

Given the vital importance of robust data in understanding the epidemiology of hypertension and diabetes amongst PLWH, a systematic review of current reporting and handling practices of missing data in this area is both timely and necessary. Therefore, we aimed in this systematic review to assess the proportion of studies that reported missing data and to examine how missing data was reported and handled in studies on co-morbid hypertension and diabetes among people living with HIV/AIDS.

## Methods

This systematic review is reported in accordance with the Preferred Reporting Items for Systematic reviews and Meta-Analysis (PRISMA) 2015 Guidelines [15]. The protocol was registered at the International Prospective Register of Systematic Review and Meta-analysis **(**PROSPERO: CRD42023391568**)**.

### Search strategy

We did a comprehensive search across PubMed-MEDLINE to identify all relevant studies published between January 1990 and June 2023. The search strategy consisted of words related to prevalence, hypertension, diabetes, and HIV/AIDS (see Additional file 1). The last search date was 1 st September 2023.

### Screening and selection of studies

To be included in the review, studies had to (1) report co-morbid hypertension and/or diabetes among adults (aged 18 years or above) PLWH worldwide, (2) be cross-sectional studies, (3) published in English or French. We excluded 1) case series, case reports, reviews, clinical trials, commentaries, and editorials, and 3) studies including children and adolescents and those not performed in human participants.

We used EPPI reviewer 4.0 [16] to screen titles and abstracts and full texts. One reviewer screened titles and abstracts of all studies, while another independently reviewed the titles and abstracts of a random third of studies retrieved from electronic searches. Two team members independently reviewed all studies included for full text screening. Disagreements were resolved through consensus and by consulting a third team member. The agreement between the reviewers was 90.4% for titles and abstracts and 81.9% for full text screening.

### Data extraction

Data were extracted using a purpose-designed and piloted extraction form. Two reviewers independently extracted data from the included studies, and any inconsistencies or disagreements were resolved by consensus or through consultation with a third reviewer.

The extracted data on study characteristics included the author’s name, year of publication, country, sample size, and data source (questionnaire and/or patient records). Regarding missing data outcomes, we noted whether the missing data and the amount of missing data were reported, the reasons for missing data, the sections of the paper and the variables for which missing data were reported, and the pattern of missingness. We also recorded whether the studies were transparent in their reporting, if they acknowledged potential biases, and whether the missing data affected the study’s conclusions. Additionally, we noted whether a sensitivity analysis was conducted and the methods used for such analysis. Finally, we documented the methods used for handling missing data, and in cases where multiple imputation was employed, we recorded the software used.

### Data analysis

We calculated proportions for each category and compared them based on the reporting of missing data status, using Chi-square and Fisher’s exact tests. Statistical significance was determined at a p-value < 0.05.

## Results

### Summary of searches and study selection

**Fig. 1:**
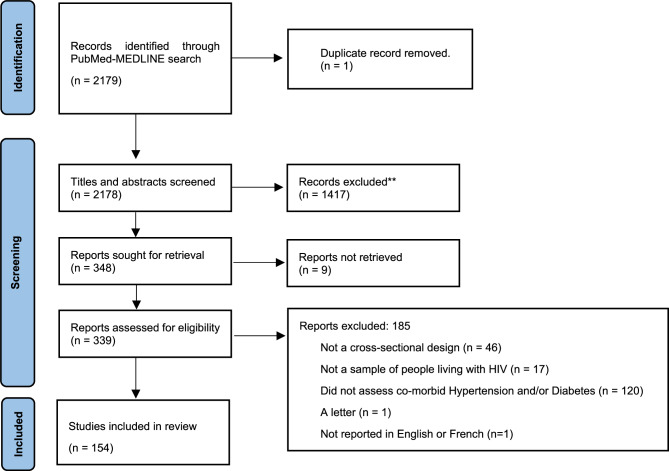
PRISMA flow diagram showing the selection process of studies included and excluded in the review

The study selection process is summarized in Fig. 1. In total, 2179 studies were identified via database searches. After deduplication, we screened the title and abstracts of 2178 articles, of which 348 were retrieved for full text screening. Of these, 154 articles met the inclusion criteria and were included in this review (see Additional file 2).

### Characteristics of included studies

The general characteristics of included studies are summarised in Table 1. Most studies were published after the year 2015, [*n* = 111, (72,1%)], and were conducted in High-income countries, 56 (36.4%). The commonest data source was questionnaires (69 studies; 44.8%). Of those using questionnaires only, 4 (2.6%) reported that the questionnaire was self-administered. The sample size of the studies ranged from 39 [17] to 9,950,296 [18], with a majority of the studies (63%) having a sample size of less than 1000 participants.

**Table 1:** Characteristics of included studies

Variable	Total *N* (%) *N* = 154	Missing data reported				*p*-value
		No, *n* = 101 *N* (%)	Yes, *n* = 53 *N* (%)			
Year of publication					0.319	
> 2015	111 (72.1)	69 (62.2)	42 (37.8)			
2011–2015	30 (19.5)	23 (76.7)	7 (23.3)			
2003–2010	13 (8.4)	9 (69.2)	4 (30.8)			
World bank classification of countries					0.106	
High income	56 (36.4)	34 (60.7)	22 (39.3)			
Upper-middle income	28 (18.2)	24 (85.7)	4 (14.3)			
Lower-middle-income	47 (30.5)	28 (59.6)	19 (40.4)			
Low income	19 (12.3)	13 (68.4)	6 (31.6)			
Multi regional	2 (1.3)	2 (100)	2 (100)			
Data source					0.079	
Both questionnaire and patient record	45 (29.2)	31 (68.9)	14 (31.1)			
Patient records only	36 (23.4)	20 (55.6)	16 (44.4)			
Questionnaire only	69 (44.8)	46 (66.7)	23 (33.3)			
Unclear	4 (2.6)	4 (100)	0 (0)			
If questionnaire, self-administered or not					0.390	
Yes	4 (2.6)	2 (50)	2 (50)			
No	75 (48.7)	51 (68)	25 (33.3)			
Unclear	39 (25.3)	29 (74.4)	10 (25.6)			
NA	36 (23.4)	20 (55.6)	16 (44.4)			
Sample size					0.011	
> 10 000	8 (5.2)	4 (50)	4 (50)			
1 000–10 000	47 (30.5)	23 (48.9)	24 (51.1)			
< 1 000	97 (63)	73 (75.3)	24 (24.7)			
Not reported	2 (1.3)	1 (50)	1 (50)			

*NA* Not Applicable

### Reporting of missing data

The distribution of missing data and the respective handling methods across the reviewed studies are detailed in Table 2. Out of the 154 studies included, 53 (34.4%) reported missing data. Of these, 30 (56.1%) stated the amount of missing data, and 19 (37.7%) studies discussed the reasons for missingness. The commonest reasons cited for missing data were: undocumented in 13 (24,6%) studies [19–28], and non-response in 4 (7.6%) studies [29–32]. Missing data was most reported in the results section of 35 studies (66.0%), and for exposure variables, it was also noted in the same number of studies (66.0%). Most studies reported missing data for CD4 count (12 studies; 22.6%) and viral load (9 studies; 17%) variables. For these variables, the percentage of missing data ranged from 0.42% [22] to 53.6% [33] for CD4 count, and from 0.40% [34] to 68% [35] for viral load.

**Table 2:** Reporting and handling of missing data in the reviewed articles

Variables	Frequency	Percentage	
	*n* = 53	(%)	
Reported amount of missing data			
No	23	43.4	
Yes	30	56.6	
Reasons for missing data			
Non-response	4	7.6	
Poor turn-up in the next day of the interview	1	1.9	
Underreporting by providers	1	1.9	
Undocumented	13	24.6	
Not reported	33	62.3	
Missing data domain			
Exposure	35	66.0	
Confounder	6	11.3	
Outcome	8	15.1	
Section in paper where missing data is reported			
Methods	22	41.5	
Results	35	66.0	
Discussion	6	12.2	
Supplementary sheet	1	1.9	
Limitations section	4	7.6	
Reported missing data for variables of interest			
WHO stage	1	1.9	
CD4 count	12	22.6	
Viral load	9	17	
Duration on ART	5	9.4	
Diabetes mellitus	1	1.9	
Hypertension	1	1.9	
Transparency in reporting of missing data			
No	26	49.1	
Yes	27	50.9	
Potential biases reported?			
No	41	77.4	
Yes	12	22.6	
Whether missing data affected conclusions			
No	13	24.5	
Not reported	40	75.5	
Sensitivity analysis for missing data done			
No	44	83	
Yes	9	17	
Sensitivity analysis methods used			
Bayesian analysis	1	1.9	
Adjusting neighbourhood cut points	1	1.9	
Excluding participants	1	1.9	
Missing data were included	1	1.9	
Propensity score methods	1	1.9	
Not applicable	44	83	
Not reported	4	7.6	
Pattern of missing data			
MCAR	0	0	
MAR	5	9.4	
MNAR	0	0	
Not reported	48	90.6	
Method for handling missing data			
Complete-case analysis	16	30.2	
Linear interpolation	1	1.9	
Multiple Imputation	5	9.4	
Single imputation	1	1.9	
list wise deletion	1	1.9	
Not reported	28	52.8	
Unclear	1	1.9	
MI: imputation software used			
Not reported	2	3.8	
SAS vs. 9.3	1	1.9	
STATA vs. 12.1	1	1.9	
STATA vs. 15.0	1	1.9	
STATA	1	1.9	

*MI* Multiple imputation, *MCAR* Missing Completely at Random, *MAR* Missing at Random, *MNAR* Missing Not at Random, *WHO* World Health Organisation

Amongst studies that reported missing data, 27 (50.9%) were transparent in their reporting. Twelve studies (22.6%) acknowledged that missing data could have biased their results. Four of these studies specified how missing data could have influenced the outcomes: one study reported the inability to assess the impact of obesity due to missing weight data [31], another cited reduced external validity [21], a third faced difficulties in analysing risk factor clustering [24], and a fourth experienced limitations in certain analyses due to reduced sample sizes [25]. Additionally, there was a concern about the underestimation of cardiometabolic disease prevalence in another study [36].

Sensitivity analysis was performed in only 9 studies (17%). The following methods were used: Bayesian analysis [23], adjusted neighbourhood cut-points [29], “exclusion of participants” [37], “inclusion of incomplete data” [37], and propensity score methods [38]. No study reported that missing data had an impact on its conclusions.

### Handling missing data

Only 24 (39%) studies reported the method used to handle missing data. Among these, 16 studies (30.2%) utilized complete case analysis [20, 21, 28, 31, 32, 35, 39–48]. Five studies (9.4%) employed multiple imputations [37, 49–52] using SAS version 9.3 [29] and STATA versions 12.1 [49] and 15.0 [37] software. One study reported handling missing data using the linear interpolation method [23]. Twenty-eight studies did not report how they handled missing data in their analysis. For studies that used multiple imputations, further details about the imputation process were often omitted, such as the number of imputations per variable, the number of imputed variables, and the statistical software used (Table 2). MAR was the only suggested pattern of missing data, reported in 5 (9.4%) studies.

## Discussion

This review is the first to systematically investigate the reporting and handling of missing data in cross-sectional studies of hypertension and diabetes among PLWH. Recognizing the importance of accurate data in epidemiological research, we assessed current practices and identified potential gaps, aiming to offer insights for future research in this important area. We found that only 34.4% of studies reported missing data. Missingness was mostly in the exposure variables, notably, CD4 count and viral load. Few studies discussed how missingness biased results and conclusions. Of the studies that reported missing data, less than half of these studies reported how they handled missing data, and for those that did, they largely used complete case analysis followed by multiple imputation methods.

The proportion of studies reporting missing data in our study aligns closely with the 37.5% reported by Masconi et al. [9] in their review on predictive research for prevalent undiagnosed type 2 diabetes mellitus. However, our figure is substantially lower than the 56% seen in multi-database pharmacoepidemiologic studies [53] and the striking 93% in non-inferiority and equivalence trials [54]. A potential reason for our lower prevalence could be that certain studies, while not having any missing data, did not explicitly confirm its absence, as recommended by the STROBE guidelines [55]. Alternatively, they may have addressed the missing data but failed to document their approach. Disparities in missing data reporting across research domains may also arise from distinct reporting standards and challenges inherent to each domain. For instance, the multifaceted nature of data collection in multi-database studies or the stringent data and reporting prerequisites in trials may account for their higher percentages of missing data compared to other research areas.

The key reasons for missingness in the 19 (37.7%) studies that reported them in our review were lack of documentation of missing data and non-response. Unlike our study, another review on missing data in palliative care trials reported up to 71% of studies indicating reasons for missingness [56]. Their main reported reasons for missingness were loss to follow-up or withdrawal. Some reasons identified by Masconi et al. were study design, participant and measurements characteristics, data collection and management and chance [9]. This points towards possible inefficiencies in data collection or reporting mechanisms. Non-responses might stem from various reasons, such as participant disinterest, survey design flaws, or logistical challenges, which could be addressed in future studies. That the most common data missing in this review were HIV related factors of CD4 count and viral load is not surprising. The missing data on CD4 count and viral load could stem from various factors, including scarce medical facilities capable of administering these tests, a lack of qualified personnel, the expense of frequent blood tests, difficulties in specimen transport, and insufficient coverage by insurance or public health systems, all of which contribute to reduced testing and reporting frequency, particularly in resource-limited settings. Additionally, the operationalized definitions of these variables may have further contributed to their missingness. For instance, if a questionnaire specified a reference window of the prior three months, but a patient’s most recent CD4 count was measured six months ago due to their clinic visit schedule, this misalignment could result in missing data. Recognizing these variables as especially prone to missingness is essential for devising strategies to enhance data completeness in future research.

While over half of the studies were transparent in their reporting, only 22.6% admitted that missing data may have introduced biases. Acknowledging such biases is fundamental to maintaining the integrity and reliability of research findings. This is particularly relevant in studies of HIV-related factors and their associations with cardiometabolic conditions, where missingness can obscure key patterns and relationships. It’s commendable that a subset of these studies provided detailed insights into how missing data may have affected their findings, from limiting body mass index calculations to impacting the external validity. Such candid admissions provide a roadmap for future research to address these potential pitfalls. Yet, when compared to the acknowledgement of potential bias in 34% of studies in the multi-database pharmacoepidemiologic review [53], there’s room for improvement in the field.

Only 24 (45.3%) studies detailed how they addressed missing data in their analyses. This raises concern as a significant number of studies failed to provide clarity on their approach, potentially utilizing techniques without mentioning them in the publication. Notably, complete case analysis was the preferred method, being utilized in 16 (30.2%) of the articles. This aligns with other reviews [9, 10, 54], which also identified complete case analysis as the predominant method for addressing missing data. The popularity of this approach might stem from its simplicity and being the default in most statistical software. However, its primary drawback is the potential for biased outcomes when the missing data isn’t Missing Completely at Random (MCAR).

A more advanced strategy, multiple imputation, which estimates missing data based on existing information, was adopted by 9.4% of the studies. Though recommended for its efficacy, especially when MCAR and MAR assumptions hold, its utilization remains limited. The lack of its widespread use could be attributed to either a lack of awareness among medical researchers or the necessity for specialized statistical proficiency to execute it. Furthermore, it’s paramount for researchers to understand the nature of their missing data, as this determines the most suitable method for addressing it. Another noteworthy point is the prevalent omission of essential details about the imputation process, such as the number of imputations per variable, the statistical assumptions underpinning the method, and the chosen statistical software. These omissions hinder the ability to critically evaluate the validity of the imputation approach and its influence on the study’s findings. Furthermore, without clear documentation of how missing data were addressed, it becomes challenging to assess the study’s robustness and reproducibility. This issue was similarly observed in a review on predictive research for prevalent undiagnosed type 2 diabetes mellitus, which reported that none of the studies discussed the specifics of multiple imputation.

Also, important is the pattern of missing data, which guides the choice of handling method. It’s worrisome that only five studies reported their data as MAR, with the remainder neglected to specify the pattern of missingness. This omission is more than a simple oversight; it denotes a potential shortfall in the rigorous assessment of the nature of missing data mechanisms. Understanding whether data are MAR, MCAR, or Missing Not at Random (MNAR) is indispensable for determining the validity of applied imputation methods or other handling strategies. Without this fundamental step, the risk of employing inappropriate methods increases, potentially biasing study findings and undermining their reliability. Therefore, a comprehensive understanding of these patterns is indispensable for making informed decisions regarding the most appropriate techniques for addressing missing data.

## Limitation

This research, while comprehensive in its analysis, offers both significant strengths and inherent limitations that merit consideration. We included 154 studies from the literature worldwide to contribute new insights on the topic in a research area where such a review has not been previously done. However, a key limitation is our reliance solely on published data, which may not reflect the entirety of missing data practices or the true magnitude of omissions. Our review could only capture what was explicitly reported, leaving the implicit practices beyond our reach. Additionally, our search was confined to PubMed and to studies in English and French, potentially introducing selection bias and limiting the generalizability of our findings.

## Conclusion

This review highlighted inadequate reporting and handling of missing data in co-morbid hypertension and diabetes studies among PLWH. There is a pressing need for the development and dissemination of comprehensive, accessible guidelines on managing missing data. Equipping researchers with these guidelines will likely improve rigorous data handling, enhance the integrity of research outcomes, and increase confidence in the published studies.

## Supplementary Information

Supplementary Material 1.

Supplementary Material 2.

Supplementary Material 3.

## Data Availability

No datasets were generated or analysed during the current study.

## Abbreviations

AIDS: Acquired Immunodeficiency Syndrome
ART: Antiretroviral therapy
GEE: Generalized estimating equations
HIV: Human Immunodeficiency Virus
MAR: Missing at random
MCAR: Missing completely at random
MI: Multiple imputation
MNAR: Missing not at random
NA: Not applicable
PLWH: People living with HIV/AIDS
PRISMA: Preferred Reporting Items for Systematic reviews and Meta-Analysis
WHO: World Health Organization
