# Supplementary material for: Reporting and handling of missing data in published studies of co-morbid hypertension and diabetes among people living with HIV/AIDS: a systematic review
Source: BMC Med Res Methodol. 2025 Jul 30;25:180. doi: 10.1186/s12874-025-02630-1 (PMC12308936; doi:10.1186/s12874-025-02630-1)
Supplement: Supplementary file 1 — Supplementary Material 1. [file 12874_2025_2630_MOESM1_ESM.pdf]

Title: **Reporting and handling of missing data in published studies of co-morbid hypertension and diabetes among people living with HIV/AIDS: a systematic review**

Search Strategy

PubMed-MEDLINE was searched for relevant studies published between January 1990 and September 2022. The last search date was 1st September 2023.

Additional Table 1: Pub-Med Search Strategy

**Search**	**Search Terms**
1	Hypertension[mesh] OR Hypertension[tw] OR blood pressure[tw]
2	Diabetes mellitus[mesh] OR diabetes[tw]
3	Cross-Sectional Studies[mesh:noexp] OR cross-sectional[tiab] OR cross sectional[tiab] OR Prevalence[mesh:noexp] OR prevalence[tiab] OR transversal study[tiab]
4	HIV[mesh] OR HIV[tw] OR HIV/AIDS[tw] OR human immunodeficiency virus[tw] OR acquired immunodeficiency syndrome[tw] OR AIDS[tw] OR acquired immunodeficiency syndrome[mesh] OR HIV infection[tw]
5	#1 OR #2
6	#3 AND #4 AND #5
7	Review[pt] OR clinical trial[pt] OR case reports[pt] OR meta-analysis[pt]
8	#6 NOT #7
9	Filters: Humans, English, French, Publication
