# Supplementary material for: Reporting and handling of missing data in published studies of co-morbid hypertension and diabetes among people living with HIV/AIDS: a systematic review
Source: BMC Med Res Methodol. 2025 Jul 30;25:180. doi: 10.1186/s12874-025-02630-1 (PMC12308936; doi:10.1186/s12874-025-02630-1)
Supplement: Supplementary file 2 — Supplementary Material 2. [file 12874_2025_2630_MOESM2_ESM.pdf]

Additional Table 2: **Overview of studies on hypertension and diabetes mellitus prevalence with various methodologies across different countries**

Author year	Publication year	Country	Data source	Self-administered Questionnaire	Sample size	Outcome of interest	Missing data reported	Sensitivity analysis done?	Method for handling missing data
Abebe et al. (1)	2016	Ethiopia	Questionnaire	unclear	462	Diabetes Mellitus	no	NA	NA
Achwoka et al.(2)	2020	Kenya	Patient records	NA	1478	Hypertension and Diabetes Mellitus	yes	yes	unclear
Ahmed et al.(3)	2022	Kenya	Questionnaire	no	200	Hypertension and Diabetes Mellitus	yes	no	NR
Ang et al.(4)	2021	Singapore	Patient records	NA	2231	Hypertension and Diabetes Mellitus	yes	no	complete-case analysis
Antonello et al.(5)	2015	Brazil	Patient records	NA	1009	Hypertension	no	NA	NA
Appiah et al.(6)	2019	Ghana	Questionnaire	no	506	Hypertension and Diabetes Mellitus	no	NA	NA
Arruda et al.(7)	2010	Brazil	Both questionnaire and patient record	unclear	958	Hypertension	no	NA	NA
Badru et al.(8)	2022	Nigeria	Both questionnaire and patient record	no	301	Hypertension	no	NA	NA
Baekken et al.(9)	2008	Norway	Questionnaire	yes	542	Hypertension	no	NA	NA
Basil et al.(10)	2021	USA	Both questionnaire and patient record	unclear	1760	Diabetes Mellitus	no	NA	NA
Bastard et al.(11)	2019	France	Questionnaire	unclear	352	Hypertension and Diabetes Mellitus	no	NA	NA
Begovac et al.(12)	2015	Croatia and Serbia	Questionnaire	unclear	254	Hypertension and Diabetes Mellitus	no	NA	NA
Benzekri et al.(13)	2018	Senegal	Patient records	NA	2848	Hypertension	yes	no	complete-case analysis
Bergersen et al.(14)	2003	Norway	Unclear	unclear	721	Hypertension	no	NA	NA
Bernardino et al.(15)	2011	Spain	Both questionnaire and patient record	unclear	310	Hypertension	no	NA	NA
Birabaharan et al. (16)	2022	USA	Patient records	NA	39485	Diabetes Mellitus	no	NA	NA
Bloomfield et al. (17)	2011	Kenya	Patient records	NA	12194	Hypertension	yes	no	complete-case analysis
Brar et al. (18)	2007	USA	Unclear	unclear	9150	Diabetes Mellitus	no	NA	NA
Brunettaet al. (19)	2022	canada	Patient records	NA	2000	Hypertension and Diabetes Mellitus	yes	No	complete-case analysis
Buchacz et al.(20)	2013	USA	Patient records	NA	3166	Hypertension and Diabetes Mellitus	yes	no	NR
Buendia et al.(21)	2022	USA	Patient records	NA	989	Diabetes Mellitus	no	NA	NA

Burkholder et al. (22)	2018	USA	Both questionnaire and patient record	yes	1664	Hypertension	yes	no	NR
Butt et al. (23)	2009	USA	Patient records	NA	6567	Diabetes Mellitus	no	NA	NA
Calza et al.(24)	2011	Italy	Questionnaire	unclear	755	Diabetes Mellitus	no	NA	NA
Carvalho et al.(25)	2018	Brazil	Questionnaire	unclear	67	Hypertension and Diabetes Mellitus	no	NA	NA
Cechin et al. (26)	2023	UK	Both questionnaire and patient record	no	948	Diabetes Mellitus	no	NA	NA
Chepchirchir et al. (27)	2018	Kenya	Both questionnaire and patient record	no	297	Hypertension	no	NA	NA
Chhoun et al.(28)	2017	Cambodia	Questionnaire	no	510	Hypertension and Diabetes Mellitus	no	NA	NA
Chihota et al.(29)	2022	South Africa	Patient records	NA	420	Hypertension and Diabetes Mellitus	no	NA	NA
Chinaeke et al.(30)	2023	USA	Patient records	NA	2509	Diabetes Mellitus	no	NA	NA
Chireshe et al.(31)	2019	Zimbabwe	Questionnaire	no	600	Hypertension	no	NA	NA
Chiwandire et al.(32)	2021	South Africa	Questionnaire	no	4484	Hypertension and Diabetes Mellitus	no	NA	NA
Chu et al.(33)	2011	USA	Patient records	NA	854	Hypertension and Diabetes Mellitus	no	NA	NA
Ciccacci et al.(34)	2019	Malawi	Patient records	NA	7071	Hypertension and Diabetes Mellitus	no	NA	NA
Clark et al.(35)	2015	South Africa	Both questionnaire and patient record	no	3641	Hypertension and Diabetes Mellitus	no	NA	NA
Coelho et al.(36)	2018	Portugal	Questionnaire	unclear	220	Diabetes Mellitus	no	NA	NA
Coetzee et al.(37)	2019	SSA	Both questionnaire and patient record	unclear	NR	Hypertension and Diabetes Mellitus	yes	yes	Linear interpolation
Collins et al.(38)	2021	USA	Questionnaire	no	3232	Hypertension and Diabetes Mellitus	yes	no	NR
Collins et al.(39)	2023	USA	Patient records	NA	5929	Hypertension and Diabetes Mellitus	yes	no	complete-case analysis
Cope et al.(40)	2020	USA	Questionnaire	unclear	2342	Hypertension and Diabetes Mellitus	yes	yes	NR
Costa et al.(41)	2020	Brazil	Both questionnaire and patient record	unclear	298	Hypertension	no	NA	NA
Crane et al.(42)	2006	USA	Both questionnaire and patient record	unclear	1441	Diabetes Mellitus	yes	no	NR
Cunha et al.(43)	2018	Brazil	Questionnaire	no	208	Hypertension	no	NA	NA
da Cunha et al.(44)	2020	Brazil	Questionnaire	no	168	Diabetes Mellitus	no	NA	NA
Danoff et al.(45)	2005	USA	Both questionnaire and patient record	unclear	272	Diabetes Mellitus	no	NA	NA

Davis et al.(46)	2023	Zimbabze	Questionnaire	no	936	Hypertension	yes	yes	complete-case analysis
De Socio et al.(47)	2014	Italy	Unclear	unclear	1182	Hypertension	no	NA	NA
de Vries et al.(48)	2023	South Africa	Questionnaire	no	356	Diabetes Mellitus	no	NA	NA
Dejkhamron et al.(49)	2014	Thailand	Both questionnaire and patient record	no	39	Diabetes Mellitus	no	NA	NA
Deloumeaux et al.(50)	2011	France	Both questionnaire and patient record	unclear	237	Hypertension and Diabetes Mellitus	no	NA	NA
DerSarkissian et al.(51)	2020	USA	Patient records	NA	3456	Hypertension and Diabetes Mellitus	no	NA	NA
Dimala et al.(52)	2016	Cameroon	Both questionnaire and patient record	no	200	Diabetes Mellitus	no	NA	NA
Dimala et al.(53)	2016	Cameroon	Both questionnaire and patient record	no	200	Hypertension	no	NA	NA
Ding et al.(54)	2017	China	Questionnaire	unclear	345	Hypertension	no	NA	NA
Divala et al.(55)	2016	Malawi	Questionnaire	no	952	Hypertension and Diabetes Mellitus	no	NA	NA
D'Souza et al.(56)	2021	USA	Patient records	NA	338	Hypertension and Diabetes Mellitus	no	NA	NA
Ekrikpo et al.(57)	2018	Nigeria	Both questionnaire and patient record	unclear	6742	Hypertension and Diabetes Mellitus	yes	no	NR
Enriquez et al.(58)	2022	uganda	Patient records	NA	990	Hypertension and Diabetes Mellitus	no	NA	NA
Faurholt-Jepsen et al. (59)	2019	Ethiopia	Both questionnaire and patient record	no	453	Diabetes Mellitus	yes	no	NR
Ferrara et al.(60)	2014	USA	Both questionnaire and patient record	no	2229	Diabetes Mellitus	yes	no	NR
Fiseha et al.(61)	2019	Ethiopia	Questionnaire	no	408	Diabetes Mellitus	no	NA	NA
Fontela et al.(62)	2018	Spain	Patient records	NA	339	Hypertension and Diabetes Mellitus	no	NA	NA
Freitas et al. (63)	2012	Caucasus	Questionnaire	unclear	364	Hypertension	no	NA	NA
Galaviz et al.(64)	2020	USA	Patient records	NA	233	Diabetes Mellitus	no	NA	NA
Galli et al.(65)	2012	Italy	Questionnaire	no	4249	Diabetes Mellitus	no	NA	NA
Gebrie et al.(66)	2020	Ethiopia	Both questionnaire and patient record	unclear	407	Hypertension	no	NA	NA
Geldsetzer et al.(67)	2017	South Africa	Questionnaire	unclear	7608	Hypertension	no	NA	NA
George et al.(68)	2019	South Africa	Both questionnaire and patient record	unclear	330	Hypertension and Diabetes Mellitus	yes	yes	NR
Gianotti et al.(69)	2011	Italy	Questionnaire	yes	99	Diabetes Mellitus	yes	no	NR
Glass et al.(70)	2006	Switzerland	Questionnaire	unclear	8033	Diabetes Mellitus	yes	no	NR

Gonah et al.(71)	2020	Zimbabwe	Questionnaire	no	18233	Hypertension and Diabetes Mellitus	no	NA	NA
González-Tomé et al.(72)	2008	Spain	Questionnaire	unclear	669	Diabetes Mellitus	no	NA	NA
Guo et al.(73)	2017	China	Questionnaire	no	973	Hypertension and Diabetes Mellitus	no	NA	NA
Gutierrez et al.(74)	2013	USA	Both questionnaire and patient record	unclear	12339	Hypertension and Diabetes Mellitus	no	NA	NA
Hadavandsiri et al (75)l	2023	Germany	Questionnaire	no	1173	Hypertension and Diabetes Mellitus	yes	no	Single inputation
Harimenshi et al.(76)	2022	Burundi	Questionnaire	no	1250	Hypertension	no	NA	NA
Heron et al.(77)	2019	Australia	Patient records	NA	1525180	Diabetes Mellitus	yes	no	NR
Hyde et al.(78)	2019	USA	Both questionnaire and patient record	unclear	957	Hypertension	no	NA	NA
Hyle et al.(79)	2019	South Africa	Both questionnaire and patient record	no	458	Hypertension and Diabetes Mellitus	yes	no	complete-case analysis
Idemudia et al.(80)	2018	South Africa	Questionnaire	unclear	600	Hypertension	yes	no	NR
Ikeda et al.(81)	2013	Brazil	Both questionnaire and patient record	unclear	1240	Hypertension	no	NA	NA
Isa et al.(82)	2016	Nigeria	Both questionnaire and patient record	unclear	2632	Diabetes Mellitus	no	NA	NA
Iwuala et al.(83)	2015	Nigeria	Both questionnaire and patient record	unclear	145	Hypertension	no	NA	NA
Jackson et al.(84)	2022	Nigeria	Questionnaire	no	417	Hypertension	yes	no	list wise deletion
Jain et al.(85)	2007	USA	Both questionnaire and patient record	unclear	1542	Diabetes Mellitus	yes	no	NR
Jeremiah et al.(86)	2020	Tanzania	Both questionnaire and patient record	unclear	1947	Diabetes Mellitus	no	NA	NA
Jericó et al.(87)	2005	Spain	Questionnaire	unclear	1512	Hypertension	no	NA	NA
Julius et al.(88)	2011	South Africa	Questionnaire	unclear	304	Hypertension and Diabetes Mellitus	no	NA	NA
Juma et al.(89)	2019	Kenya	Patient records	NA	1502	Hypertension and Diabetes Mellitus	yes	no	complete-case analysis
Jumare et al.(90)	2023	Nigeria	Questionnaire	no	440	Hypertension and Diabetes Mellitus	no	NA	NA
Kagaruki et al.(91)	2014	Tanzania	Questionnaire	no	671	Hypertension and Diabetes Mellitus	yes	no	NR
Kagaruki et al.(92)	2018	Tanzania	Questionnaire	no	671	Hypertension and Diabetes Mellitus	yes	no	complete-case analysis
Kakar et al.(93)	2017	Australia	Patient records	NA	188	Hypertension and Diabetes Mellitus	yes	no	NR
Kamkuemah et al.(94)	2022	South Africa	Questionnaire	no	92	Hypertension	no	NA	NA

Kansiime et al.(95)	2019	Uganda	Both questionnaire and patient record	no	387	Hypertension and Diabetes Mellitus	yes	no	NR
Kaplan et al.(96)	2020	USA	Patient records	NA	232	Hypertension and Diabetes Mellitus	yes	no	MI
Kato et al.(97)	2020	Tanzania	Questionnaire	no	612	Hypertension and Diabetes Mellitus	no	NA	NA
Kazooba et al.(98)	2017	Uganda	Questionnaire	unclear	1024	Hypertension and Diabetes Mellitus	yes	no	NR
Kilewo et al.(99)	2009	Malawi,Tanzania and Zambia	Questionnaire	no	2294	Hypertension	yes	no	NR
Kourtis et al.(100)	2009	USA	Patient records	NA	9950296	Diabetes Mellitus	no	NA	NA
Kuber et al.(101)	2021	South Africa	Patient records	NA	NR	Hypertension	no	NA	NA
Kwarisiima et al.(102)	2016	Uganda	Questionnaire	no	65544	Hypertension	yes	no	complete-case analysis
Lubega et al.(103)	2021	Uganda	Both questionnaire and patient record	no	2026	Hypertension	no	NA	NA
Lukas et al.(104)	2021	Ethiopia	Questionnaire	no	382	Hypertension	no	NA	NA
Maggi et al. (105)	2022	Italy	Patient records	NA	1094	Hypertension and Diabetes Mellitus	no	NA	NA
Magodoro et al.(106)	2016	Zimbabwe	Patient records	NA	1033	Hypertension and Diabetes Mellitus	yes	no	MI
Malaza et al.(107)	2012	South Africa	Questionnaire	no	14918	Hypertension	no	NA	NA
Malindisa et al.(108)	2023	Tanzania	Questionnaire	no	223	Hypertension and Diabetes Mellitus	yes	no	NR
Manavalan et al.(109)	2020	Tanzania	Questionnaire	no	555	Hypertension	no	NA	NA
Manavalan et al.(110)	2022	Tanzania	Questionnaire	no	91	Hypertension	no	NA	NA
Masenga et al.(111)	2019	Zambia	Questionnaire	no	234	Diabetes Mellitus	no	NA	NA
Masyuko et al.(112)	2020	Kenya	Questionnaire	no	598	Hypertension and Diabetes Mellitus	yes	no	NR
Mathabire et al.(113)	2018	Malawi	Questionnaire	no	735	Hypertension and Diabetes Mellitus	no	NA	NA
Mathebula et al.(114)	2020	South Africa	Questionnaire	no	332	Hypertension and Diabetes Mellitus	no	NA	NA
Mayer et al.(115)	2018	USA	Patient records	NA	239848	Hypertension and Diabetes Mellitus	yes	no	NR
McGettigan et al.(116)	2023	uk	Patient records	NA	2951	Hypertension and Diabetes Mellitus	no	NA	NA
Medina-Torne et al.(117)	2012	USA	Both questionnaire and patient record	no	707	Hypertension	no	NA	NA
Melo et al. (118)	2020	Brazil	Both questionnaire and patient record	no	340	Hypertension and Diabetes Mellitus	no	NA	NA

Memiah et al. (119)	2021	Tanzania	Questionnaire	no	261	Hypertension	yes	no	NR
Mogaka et al. (120)	2022	Kenya	Both questionnaire and patient record	no	598	Hypertension	no	NA	NA
Mogaka et al.(121)	2023	Kenya	Questionnaire	no	300	Hypertension	no	NA	NA
Muronya et al.(122)	2011	Malawi	Questionnaire	no	174	Hypertension and Diabetes Mellitus	no	NA	NA
Musekwa et al.(123)	2021	Zambia	Patient records	NA	348	Hypertension	no	NA	NA
Mwakyandile et al.(124)	2023	Tanzania	Both questionnaire and patient record	no	430	Hypertension	no	NA	NA
Myerson et al.(125)	2014	USA	Patient records	NA	4278	Hypertension	yes	yes	MI
Nartey et al.(126)	2023	ghana	Questionnaire	no	311	Hypertension	no	NA	NA
Ngu et al.(127)	2018	Cameroon	Both questionnaire and patient record	no	311	Hypertension and Diabetes Mellitus	no	NA	NA
Niwaha et al.(128)	2021	Uganda	Both questionnaire and patient record	no	1321	Hypertension	yes	yes	NR
Niwaha et al.(129)	2022	uganda	Questionnaire	no	140	Hypertension	yes	no	NR
Njoroge et al.(130)	2021	Kenya	Both questionnaire and patient record	no	600	Diabetes Mellitus	no	NA	NA
Ogunmola et al.(131)	2014	Nigeria	Questionnaire	no	403	Hypertension	no	NA	NA
Ojong et al.(132)	2022	Nigeria	Questionnaire	no	150	Hypertension and Diabetes Mellitus	no	NA	NA
Ottaru et al.(133)	2022	Tanzania	Questionnaire	no	629	Hypertension and Diabetes Mellitus	yes	no	complete-case analysis
Oyawa et al.(134)	2022	Kenya	Both questionnaire and patient record	no	280	Hypertension	yes	no	complete-case analysis
Peck et al.(135)	2014	Tanzania	Questionnaire	no	454	Hypertension	no	NA	NA
Pérez-Chaparro et al.(136)	2021	Germany	Patient records	NA	446	Hypertension and Diabetes Mellitus	no	NA	NA
Pierre et al.(137)	2019	Haiti	Questionnaire	no	497	Hypertension and Diabetes Mellitus	yes	yes	MI
Puhr et al.(138)	2019	Australia	Both questionnaire and patient record	yes	446	Hypertension and Diabetes Mellitus	no	NA	NA
Rajagopaul et al.(139)	2021	South Africa	Questionnaire	no	301	Hypertension and Diabetes Mellitus	yes	no	complete-case analysis
Rhee et al.(140)	2016	Cameroon	Questionnaire	no	500	Diabetes Mellitus	yes	no	complete-case analysis
Russell et al.(141)	2020	Canada	Questionnaire	no	289	Hypertension and Diabetes Mellitus	no	NA	NA
Sander et al.(142)	2015	Uganda	Both questionnaire and patient record	no	1006	Hypertension and Diabetes Mellitus	no	NA	NA

Seang et al.(143)	2022	cambodia	Questionnaire	no	370	Hypertension and Diabetes Mellitus	no	NA	NA
Serrão et al.(144)	2019	Portugal	Both questionnaire and patient record	no	401	Hypertension and Diabetes Mellitus	yes	no	complete-case analysis
Shidhaye et al.(145)	2023	india	Unclear	NR	460	Hypertension and Diabetes Mellitus	no	NA	NA
Steiniche et al.(146)	2016	Guinea-Bissau	Questionnaire	no	989	Diabetes Mellitus	yes	no	complete-case analysis
Tadesse et al.(147)	2022	Ethiopia	Both questionnaire and patient record	no	363	Diabetes Mellitus	no	NA	NA
Trifirò et al.(148)	2023	Tanzania	Patient records	NA	242	Hypertension	no	NA	NA
Tsuro et al.(149)	2022	South Africa	Questionnaire	no	361	Hypertension	no	NA	NA
van Zoest et al.(150)	2016	Netherlands	Both questionnaire and patient record	unclear	1148	Hypertension	yes	yes	MI
Wallace et al.(151)	2021	USA	Patient records	NA	5876	Diabetes Mellitus	yes	no	NR
Willig et al.(152)	2015	USA	Patient records	NA	1800	Hypertension and Diabetes Mellitus	yes	no	NR
Wu et al.(153)	2014	taiwan	Questionnaire	no	920	Hypertension and Diabetes Mellitus	no	NA	NA
Ximenes et al.(154)	2015	Brazil	Both questionnaire and patient record	no	3887	Hypertension and Diabetes Mellitus	no	NA	NA

NA = Not Applicable, NR =. Not Reported

References

1. Abebe SM, Getachew A, Fasika S, Bayisa M, Girma Demisse A, Mesfin N. Diabetes mellitus among HIV-infected individuals in follow-up care at University of Gondar Hospital, Northwest Ethiopia. BMJ Open. 2016 Aug 18;6(8):e011175.

2. Achwoka D, Oyugi JO, Mutave R, Munywoki P, Achia T, Akolo M, et al. High prevalence of non-communicable diseases among key populations enrolled at a large HIV prevention & treatment program in Kenya. PloS one. 2020;15(7):e0235606.

3. Ahmed HA, Mohamed J, Akuku IG, Lee KK, Alam SR, Perel P, et al. Cardiovascular risk factors and markers of myocardial injury and inflammation in people living with HIV in Nairobi, Kenya: a pilot cross-sectional study. BMJ Open. 2022 Jun 6;12(6):e062352.

4. Ang LW, Ng OT, Boudville IC, Leo YS, Wong CS. An observational study of the prevalence of metabolic syndrome in treatment-experienced people living with HIV in Singapore. PLoS One. 2021;16(6):e0252320.

5. Antonello VS, Antonello IC, Grossmann TK, Tovo CV, Pupo BB, Winckler Lde Q. Hypertension--an emerging cardiovascular risk factor in HIV infection. Journal of the American Society of Hypertension : JASH. 2015 May;9(5):403–7.

6. Appiah LT, Sarfo FS, Huffman MD, Nguah SB, Stiles JK. Cardiovascular risk factors among Ghanaian patients with HIV: A cross-sectional study. Clinical cardiology. 2019 Dec;42(12):1195–201.

7. Arruda Júnior ER, Lacerda HR, Moura LC, Albuquerque Mde F, Miranda Filho Dde B, Diniz GT, et al. Profile of patients with hypertension included in a cohort with HIV/AIDS in the state of Pernambuco, Brazil. Arquivos brasileiros de cardiologia. 2010 Oct;95(5):640–7.

8. Badru O, Oduola T, Abdulrazaq A, Peter C. Prevalence and Predictive Factors of Hypertension Among People Living With HIV in Kebbi State, Nigeria: A Cross-sectional Study. The Journal of the Association of Nurses in AIDS Care : JANAC. 2022 Jan;33(1):e6–14.

9. Baekken M, Os I, Sandvik L, Oektedalen O. Hypertension in an urban HIV-positive population compared with the general population: influence of combination antiretroviral therapy. Journal of hypertension. 2008 Nov;26(11):2126–33.

10. Basil RC, Brown TT, Haberlen S, Rubin LH, Plankey M, Becker JT, et al. The relationship between diabetes and depressive symptoms in men with or at risk of HIV infection. HIV medicine. 2021 Jan;22(1):37–46.

11. Bastard JP, Couffignal C, Fellahi S, Bard JM, Mentre F, Salmon D, et al. Diabetes and dyslipidaemia are associated with oxidative stress independently of inflammation in long-term antiretroviral-treated HIV-infected patients. Diabetes & metabolism. 2019 Dec;45(6):573–81.

12. Begovac J, Dragović G, Višković K, Kušić J, Perović Mihanović M, Lukas D, et al. Comparison of four international cardiovascular disease prediction models and the prevalence of eligibility for lipid lowering therapy in HIV infected patients on antiretroviral therapy. Croatian medical journal. 2015 Feb;56(1):14–23.

13. Benzekri NA, Seydi M, N Doye I, Toure M, Sy MP, Kiviat NB, et al. Increasing prevalence of hypertension among HIV-positive and negative adults in Senegal, West Africa, 1994-2015. PloS one. 2018;13(12):e0208635.

14. Bergersen BM, Sandvik L, Dunlop O, Birkeland K, Bruun JN. Prevalence of hypertension in HIV-positive patients on highly active retroviral therapy (HAART) compared with HAART-naïve and HIV-negative controls: results from a Norwegian study of 721 patients. European journal of clinical microbiology & infectious diseases : official publication of the European Society of Clinical Microbiology. 2003 Dec;22(12):731–6.

15. Bernardino JI, Mora M, Zamora FX, Arribas B, Montes ML, Pascual-Pareja F, et al. Hypertension and isolated office hypertension in HIV-infected patients determined by ambulatory blood pressure monitoring: prevalence and risk factors. Journal of acquired immune deficiency syndromes (1999). 2011 Sep;58(1):54–9.

16. Birabaharan M, Strunk A, Kaelber DC, Smith DM, Martin TCS. Sex differences in type 2 diabetes mellitus prevalence among persons with HIV. AIDS. 2022 Mar 1;36(3):383–9.

17. Bloomfield GS, Hogan JW, Keter A, Sang E, Carter EJ, Velazquez EJ, et al. Hypertension and obesity as cardiovascular risk factors among HIV seropositive patients in Western Kenya. PloS one. 2011;6(7):e22288.

18. Brar I, Shuter J, Thomas A, Daniels E, Absalon J. A comparison of factors associated with prevalent diabetes mellitus among HIV-Infected antiretroviral-naive individuals versus individuals in the National Health and Nutritional Examination Survey cohort. Journal of acquired immune deficiency syndromes (1999). 2007 May;45(1):66–71.

19. Brunetta JM, Baril JG, de Wet JJ, Fraser C, Rubin G, Thomas R, et al. Cross-sectional comparison of age- and gender-related comorbidities in people living with HIV in Canada. Medicine (Baltimore). 2022 Jul 15;101(28):e29850.

20. Buchacz K, Baker RK, Palella FJ Jr, Shaw L, Patel P, Lichtenstein KA, et al. Disparities in prevalence of key chronic diseases by gender and race/ethnicity among antiretroviral-treated HIV-infected adults in the US. Antiviral therapy. 2013;18(1):65–75.

21. Buendia JR, Sears S, Griffin E, Mgbere OO. Prevalence and risk factors of type II diabetes mellitus among people living with HIV in Texas. AIDS Care. 2022 Jul;34(7):900–7.

22. Burkholder GA, Tamhane AR, Safford MM, Muntner PM, Willig AL, Willig JH, et al. Racial disparities in the prevalence and control of hypertension among a cohort of HIV-infected patients in the southeastern United States. PloS one. 2018;13(3):e0194940.

23. Butt AA, McGinnis K, Rodriguez-Barradas MC, Crystal S, Simberkoff M, Goetz MB, et al. HIV infection and the risk of diabetes mellitus. AIDS (London, England). 2009 Jun;23(10):1227–34.

24. Calza L, Masetti G, Piergentili B, Trapani F, Cascavilla A, Manfredi R, et al. Prevalence of diabetes mellitus, hyperinsulinaemia and metabolic syndrome among 755 adult patients with HIV-1 infection. International journal of STD & AIDS. 2011 Jan;22(1):43–5.

25. Carvalho PVDC, Caporali JFM, Vieira ÉLM, Guimarães NS, Fonseca MO, Tupinambás U. Evaluation of inflammatory biomarkers, carotid intima-media thickness and cardiovascular risk in HIV-1 treatment-naive patients. Revista da Sociedade Brasileira de Medicina Tropical. 2018 May;51(3):277–83.

26. Cechin L, Campbell L, Oliveira A, Goff LM, Post FA. HbA1c screening for diabetes mellitus and to evaluate diabetic control in people of African ancestry with HIV in South London. Int J STD AIDS. 2023 Jun;34(7):484–7.

27. Chepchirchir A, Jaoko W, Nyagol J. Risk indicators and effects of hypertension on HIV/AIDS disease progression among patients seen at Kenyatta hospital HIV care center. AIDS care. 2018 May;30(5):544–50.

28. Chhoun P, Ngin C, Tuot S, Pal K, Steel M, Dionisio J, et al. Non-communicable diseases and related risk behaviors among men and women living with HIV in Cambodia: findings from a cross-sectional study. International journal for equity in health. 2017 Jul;16(1):125.

29. Chihota BV, Riebensahm C, Muula G, Sinkala E, Chilengi R, Mulenga L, et al. Liver steatosis and metabolic dysfunction-associated fatty liver disease among HIV-positive and negative adults in urban Zambia. BMJ Open Gastroenterol. 2022 Jul;9(1).

30. Chinaeke EE, Li M, Love BL, Bookstaver B, Li X, Reeder G, et al. Economic impact of comorbid diabetes and associated racial disparities in managing Medicare beneficiaries with human immunodeficiency virus/acquired immune deficiency syndrome (HIV/AIDS). AIDS Care. 2023 Aug;35(8):1076–82.

31. Chireshe R, Naidoo K, Nyamakura R. Hypertension among human immunodeficiency virus infected patients on treatment at Parirenyatwa Hospital: A descriptive study. African journal of primary health care & family medicine. 2019 Aug;11(1):e1–8.

32. Chiwandire N, Zungu N, Mabaso M, Chasela C. Trends, prevalence and factors associated with hypertension and diabetes among South African adults living with HIV, 2005-2017. BMC public health. 2021 Mar;21(1):462.

33. Chu C, Umanski G, Blank A, Meissner P, Grossberg R, Selwyn PA. Comorbidity-related treatment outcomes among HIV-infected adults in the Bronx, NY. Journal of urban health : bulletin of the New York Academy of Medicine. 2011 Jun;88(3):507–16.

34. Ciccacci F, Tolno VT, Doro Altan AM, Liotta G, Orlando S, Mancinelli S, et al. Noncommunicable Diseases Burden and Risk Factors in a Cohort of HIV+ Elderly Patients in Malawi. AIDS research and human retroviruses. 2019 Nov;35(11–12):1106–11.

35. Clark SJ, Gómez-Olivé FX, Houle B, Thorogood M, Klipstein-Grobusch K, Angotti N, et al. Cardiometabolic disease risk and HIV status in rural South Africa: establishing a baseline. BMC public health. 2015 Feb;15:135.

36. Coelho AR, Moreira FA, Santos AC, Silva-Pinto A, Sarmento A, Carvalho D, et al. Diabetes mellitus in HIV-infected patients: fasting glucose, A1c, or oral glucose tolerance test - which method to choose for the diagnosis? BMC infectious diseases. 2018 Jul;18(1):309.

37. Coetzee L, Bogler L, De Neve JW, Bärnighausen T, Geldsetzer P, Vollmer S. HIV, antiretroviral therapy and non-communicable diseases in sub-Saharan Africa: empirical evidence from 44 countries over the period 2000 to 2016. Journal of the International AIDS Society. 2019 Jul;22(7):e25364.

38. Collins LF, Sheth AN, Mehta CC, Naggie S, Golub ET, Anastos K, et al. The Prevalence and Burden of Non-AIDS Comorbidities Among Women Living With or at Risk for Human Immunodeficiency Virus Infection in the United States. Clinical infectious diseases : an official publication of the Infectious Diseases Society of America. 2021 Apr;72(8):1301–11.

39. Collins LF, Palella FJJ, Mehta CC, Holloway J, Stosor V, Lake JE, et al. Aging-Related Comorbidity Burden Among Women and Men With or At-Risk for HIV in the US, 2008-2019. JAMA Netw Open. 2023 Aug 1;6(8):e2327584.

40. Cope AB, Edmonds A, Ludema C, Cole SR, Eron JJ, Anastos K, et al. Neighborhood Poverty and Control of HIV, Hypertension, and Diabetes in the Women's Interagency HIV Study. AIDS and behavior. 2020 Jul;24(7):2033–44.

41. Costa AN, Val F, Macedo ÁE, Cubas-Vega N, Tejo PLD, Marques MM, et al. Increased prevalence of hypertension among people living with HIV: where to begin? Revista da Sociedade Brasileira de Medicina Tropical. 2020;53:e20190564.

42. Crane HM, Kadane JB, Crane PK, Kitahata MM. Diabetes case identification methods applied to electronic medical record systems: their use in HIV-infected patients. Current HIV research. 2006 Jan;4(1):97–106.

43. Cunha GHD, Lima MAC, Galvão MTG, Fechine FV, Fontenele MSM, Siqueira LR. Prevalence of arterial hypertension and risk factors among people with acquired immunodeficiency syndrome. Revista latino-americana de enfermagem. 2018 Oct;26:e3066.

44. da Cunha GH, Franco KB, Galvão MTG, Lima MAC, Fontenele MSM, Siqueira LR, et al. Diabetes mellitus in people living with HIV/AIDS: prevalence and associated risk factors. AIDS care. 2020 May;32(5):600–7.

45. Danoff A, Shi Q, Justman J, Mulligan K, Hessol N, Robison E, et al. Oral glucose tolerance and insulin sensitivity are unaffected by HIV infection or antiretroviral therapy in overweight women. Journal of acquired immune deficiency syndromes (1999). 2005 May;39(1):55–62.

46. Davis K, Moorhouse L, Maswera R, Mandizvidza P, Dadirai T, Museka T, et al. Associations between HIV status and self-reported hypertension in a high HIV prevalence sub-Saharan African population: a cross-sectional study. BMJ Open. 2023 Jan 12;13(1):e067327.

47. De Socio GV, Ricci E, Maggi P, Parruti G, Pucci G, Di Biagio A, et al. Prevalence, awareness, treatment, and control rate of hypertension in HIV-infected patients: the HIV-HY study. American journal of hypertension. 2014 Feb;27(2):222–8.

48. de Vries AEM, Xaba Z, Moraba SR, Goerlitz L, Tempelman HA, Klipstein-Grobusch K, et al. Unmasking a silent killer: Prevalence of diagnosed and undiagnosed diabetes mellitus among people living with HIV in rural South Africa. Trop Med Int Health. 2023 May;28(5):367–73.

49. Dejkhamron P, Unachak K, Aurpibul L, Sirisanthana V. Insulin resistance and lipid profiles in HIV-infected Thai children receiving lopinavir/ritonavir-based highly active antiretroviral therapy. Journal of pediatric endocrinology & metabolism : JPEM. 2014 May;27(5–6):403–12.

50. Deloumeaux J, Maachi M, Sow-Goerger MT, Lamaury I, Velayoudom FL, Cheret A, et al. Adiponectin and leptin in Afro-Caribbean men and women with HIV infection: association with insulin resistance and type 2 diabetes. Diabetes & metabolism. 2011 Apr;37(2):98–104.

51. DerSarkissian M, Bhak RH, Oglesby A, Priest J, Gao E, Macheca M, et al. Retrospective analysis of comorbidities and treatment burden among patients with HIV infection in a US Medicaid population. Current medical research and opinion. 2020 May;36(5):781–8.

52. Dimala CA, Atashili J, Mbuagbaw JC, Wilfred A, Monekosso GL. A Comparison of the Diabetes Risk Score in HIV/AIDS Patients on Highly Active Antiretroviral Therapy (HAART) and HAART-Naïve Patients at the Limbe Regional Hospital, Cameroon. PloS one. 2016;11(5):e0155560.

53. Dimala CA, Atashili J, Mbuagbaw JC, Wilfred A, Monekosso GL. Prevalence of Hypertension in HIV/AIDS Patients on Highly Active Antiretroviral Therapy (HAART) Compared with HAART-Naïve Patients at the Limbe Regional Hospital, Cameroon. PloS one. 2016;11(2):e0148100.

54. Ding Y, Lin H, Liu X, Zhang Y, Wong FY, Sun YV, et al. Hypertension in HIV-Infected Adults Compared with Similar but Uninfected Adults in China: Body Mass Index-Dependent Effects of Nadir CD4 Count. AIDS research and human retroviruses. 2017 Nov;33(11):1117–25.

55. Divala OH, Amberbir A, Ismail Z, Beyene T, Garone D, Pfaff C, et al. The burden of hypertension, diabetes mellitus, and cardiovascular risk factors among adult Malawians in HIV care: consequences for integrated services. BMC public health. 2016 Dec;16(1):1243.

56. D'Souza G, Benning L, Stosor V, Witt MD, Johnson J, Friedman M, et al. The shifting picture of HIV treatment, comorbidity and substance use among US MSM living with HIV. HIV Med. 2021 Aug;22(7):538–46.

57. Ekrikpo UE, Akpan EE, Ekott JU, Bello AK, Okpechi IG, Kengne AP. Prevalence and correlates of traditional risk factors for cardiovascular disease in a Nigerian ART-naive HIV population: a cross-sectional study. BMJ open. 2018 Jul;8(7):e019664.

58. Enriquez R, Ssekubugu R, Ndyanabo A, Marrone G, Gigante B, Chang LW, et al. Prevalence of cardiovascular risk factors by HIV status in a population-based cohort in South Central Uganda: a cross-sectional survey. J Int AIDS Soc. 2022 Apr;25(4):e25901.

59. Faurholt-Jepsen D, Olsen MF, Andersen AB, Kæstel P, Abdissa A, Amare H, et al. Hyperglycemia and insulin function in antiretroviral treatment-naive HIV patients in Ethiopia: a potential new entity of diabetes in HIV? AIDS (London, England). 2019 Aug;33(10):1595–602.

60. Ferrara M, Umlauf A, Sanders C, Meyer JM, Allen McCutchan J, Duarte N, et al. The concomitant use of second-generation antipsychotics and long-term antiretroviral therapy may be associated with increased cardiovascular risk. Psychiatry research. 2014 Aug;218(1–2):201–8.

61. Fiseha T, Belete AG. Diabetes mellitus and its associated factors among human immunodeficiency virus-infected patients on anti-retroviral therapy in Northeast Ethiopia. BMC research notes. 2019 Jul;12(1):372.

62. Fontela C, Castilla J, Juanbeltz R, Martínez-Baz I, Rivero M, O'Leary A, et al. Comorbidities and cardiovascular risk factors in an aged cohort of HIV-infected patients on antiretroviral treatment in a Spanish hospital in 2016. Postgraduate medicine. 2018 Apr;130(3):317–24.

63. Freitas P, Carvalho D, Santos AC, Madureira AJ, Xerinda S, Martinez E, et al. Central/Peripheral fat mass ratio is associated with increased risk of hypertension in HIV-infected patients. Journal of clinical hypertension (Greenwich, Conn). 2012 Sep;14(9):593–600.

64. Galaviz KI, Varughese R, Agan BK, Marconi VC, Chu X, Won SH, et al. The Intersection of HIV, Diabetes, and Race: Exploring Disparities in Diabetes Care among People Living with HIV. Journal of the International Association of Providers of AIDS Care. 2020 Jan;19:2325958220904241.

65. Galli L, Salpietro S, Pellicciotta G, Galliani A, Piatti P, Hasson H, et al. Risk of type 2 diabetes among HIV-infected and healthy subjects in Italy. European journal of epidemiology. 2012 Aug;27(8):657–65.

66. Gebrie A. Hypertension among people living with human immunodeficiency virus receiving care at referral hospitals of Northwest Ethiopia: A cross-sectional study. PloS one. 2020;15(8):e0238114.

67. Geldsetzer P, Feigl AB, Tanser F, Gareta D, Pillay D, Bärnighausen T. Population-level decline in BMI and systolic blood pressure following mass HIV treatment: Evidence from rural KwaZulu-Natal. Obesity (Silver Spring, Md). 2017 Jan;25(1):200–6.

68. George S, McGrath N, Oni T. The association between a detectable HIV viral load and non-communicable diseases comorbidity in HIV positive adults on antiretroviral therapy in Western Cape, South Africa. BMC infectious diseases. 2019 Apr;19(1):348.

69. Gianotti N, Visco F, Galli L, Barda B, Piatti P, Salpietro S, et al. Detecting impaired glucose tolerance or type 2 diabetes mellitus by means of an oral glucose tolerance test in HIV-infected patients. HIV medicine. 2011 Feb;12(2):109–17.

70. Glass TR, Ungsedhapand C, Wolbers M, Weber R, Vernazza PL, Rickenbach M, et al. Prevalence of risk factors for cardiovascular disease in HIV-infected patients over time: the Swiss HIV Cohort Study. HIV medicine. 2006 Sep;7(6):404–10.

71. Gonah L, Moodley I, Hlongwana K. Prevalence of diabetes mellitus and hypertension in people living with human immunodeficiency virus on antiretroviral therapy in Gweru district, Zimbabwe. African journal of primary health care & family medicine. 2020 Aug;12(1):e1–6.

72. González-Tomé MI, Ramos Amador JT, Guillen S, Solís I, Fernández-Ibieta M, Muñoz E, et al. Gestational diabetes mellitus in a cohort of HIV-1 infected women. HIV medicine. 2008 Nov;9(10):868–74.

73. Guo F, Hsieh E, Lv W, Han Y, Xie J, Li Y, et al. Cardiovascular disease risk among Chinese antiretroviral-naïve adults with advanced HIV disease. BMC infectious diseases. 2017 Apr;17(1):287.

74. Gutierrez J, Elkind MS, Marshall RS. Cardiovascular profile and events of US adults 20-49 years with HIV: results from the NHANES 1999-2008. AIDS care. 2013;25(11):1385–91.

75. Hadavandsiri F, Shafaati M, Mohammad Nejad S, Ebrahimzadeh Mousavi M, Najafi A, Mirzaei M, et al. Non-communicable disease comorbidities in HIV patients: diabetes, hypertension, heart disease, and obstructive sleep apnea as a neglected issue. Sci Rep. 2023 Aug 5;13(1):12730.

76. Harimenshi D, Niyongabo T, Preux PM, Aboyans V, Desormais I. Hypertension and associated factors in HIV-infected patients receiving antiretroviral treatment in Burundi: a cross-sectional study. Sci Rep. 2022 Nov 28;12(1):20509.

77. Heron JE, Norman SM, Yoo J, Lembke K, O'Connor CC, Weston CE, et al. The prevalence and risk of non-infectious comorbidities in HIV-infected and non-HIV infected men attending general practice in Australia. PloS one. 2019;14(10):e0223224.

78. Hyde JR, Sears SC, Buendia JR, Odem SL, Vaaler ML, Mgbere OO. HIV Comorbidities-Pay Attention to Hypertension Amid Changing Guidelines: An Analysis of Texas Medical Monitoring Project Data. American journal of hypertension. 2019 Sep;32(10):960–7.

79. Hyle EP, Bekker LG, Martey EB, Huang M, Xu A, Parker RA, et al. Cardiovascular risk factors among ART-experienced people with HIV in South Africa. Journal of the International AIDS Society. 2019 Apr;22(4):e25274.

80. Idemudia ES, Olasupo MO, Modibo MW. Stigma and chronic illness: A comparative study of people living with HIV and/or AIDS and people living with hypertension in Limpopo Province, South Africa. Curationis. 2018 Oct;41(1):e1–5.

81. Ikeda ML, Barcellos NT, Alencastro PR, Wolff FH, Brandão AB, Fuchs FD, et al. Association of blood pressure and hypertension with alcohol consumption in HIV-infected white and nonwhite patients. TheScientificWorldJournal. 2013;2013:169825.

82. Isa SE, Oche AO, Kang'ombe AR, Okopi JA, Idoko JA, Cuevas LE, et al. Human Immunodeficiency Virus and Risk of Type 2 Diabetes in a Large Adult Cohort in Jos, Nigeria. Clinical infectious diseases : an official publication of the Infectious Diseases Society of America. 2016 Sep;63(6):830–5.

83. Iwuala SO, Lesi OA, Olamoyegun MA, Sabir AA, Fasanmade OA. Lipoatrophy among patients on antiretroviral therapy in Lagos, Nigeria: Prevalence, pattern and association with cardiovascular risk factors. Nigerian journal of clinical practice. 2015 Sep;18(5):626–32.

84. Jackson IL, Lawrence SM, Igwe CN, Ukwe CV, Okonta MJ. Prevalence and control of hypertension among people living with HIV receiving care at a Nigerian hospital. Pan Afr Med J. 2022;41:153.

85. Jain MK, Aragaki C, Fischbach L, Gibson S, Arora R, May L, et al. Hepatitis C is associated with type 2 diabetes mellitus in HIV-infected persons without traditional risk factors. HIV medicine. 2007 Nov;8(8):491–7.

86. Jeremiah K, Filteau S, Faurholt-Jepsen D, Kitilya B, Kavishe BB, Krogh-Madsen R, et al. Diabetes prevalence by HbA1c and oral glucose tolerance test among HIV-infected and uninfected Tanzanian adults. PloS one. 2020;15(4):e0230723.

87. Jericó C, Knobel H, Montero M, Sorli ML, Guelar A, Gimeno JL, et al. Hypertension in HIV-infected patients: prevalence and related factors. American journal of hypertension. 2005 Nov;18(11):1396–401.

88. Julius H, Basu D, Ricci E, Wing J, Basu JK, Pocaterra D, et al. The burden of metabolic diseases amongst HIV positive patients on HAART attending The Johannesburg Hospital. Current HIV research. 2011 Jun;9(4):247–52.

89. Juma K, Nyabera R, Mbugua S, Odinya G, Jowi J, Ngunga M, et al. Cardiovascular risk factors among people living with HIV in rural Kenya: a clinic-based study. Cardiovascular journal of Africa. 2019 Jan;30(1):52–6.

90. Jumare J, Dakum P, Sam-Agudu N, Memiah P, Nowak R, Bada F, et al. Prevalence and characteristics of metabolic syndrome and its components among adults living with and without HIV in Nigeria: a single-center study. BMC Endocr Disord. 2023 Jul 28;23(1):160.

91. Kagaruki GB, Mayige MT, Ngadaya ES, Kimaro GD, Kalinga AK, Kilale AM, et al. Magnitude and risk factors of non-communicable diseases among people living with HIV in Tanzania: a cross sectional study from Mbeya and Dar es Salaam regions. BMC public health. 2014 Sep;14:904.

92. Kagaruki GB, Mayige MT, Ngadaya ES, Kilale AM, Kahwa A, Shao AF, et al. Knowledge and perception on type2 diabetes and hypertension among HIV clients utilizing care and treatment services: a cross sectional study from Mbeya and Dar es Salaam regions in Tanzania. BMC public health. 2018 Jul;18(1):928.

93. Kakar S, Drak D, Amin T, Cheung J, O'Connor C, Gracey D. Screening and management of risk factors for cardiovascular disease in HIV-positive patients attending an Australian urban sexual health clinic. Sexual health. 2017 Apr;14(2):198–200.

94. Kamkuemah M, Gausi B, Oni T. High prevalence of multimorbidity and non-communicable disease risk factors in South African adolescents and youth living with HIV: Implications for integrated prevention. S Afr Med J. 2022 Apr 4;112(4):259–67.

95. Kansiime S, Mwesigire D, Mugerwa H. Prevalence of non-communicable diseases among HIV positive patients on antiretroviral therapy at joint clinical research centre, Lubowa, Uganda. PloS one. 2019;14(8):e0221022.

96. Kaplan A, Simon TG, Henson JB, Wang T, Zheng H, Osganian SA, et al. Brief Report: Relationship Between Nonalcoholic Fatty Liver Disease and Cardiovascular Disease in Persons With HIV. Journal of acquired immune deficiency syndromes (1999). 2020 Aug;84(4):400–4.

97. Kato I, Tumaini B, Pallangyo K. Prevalence of non-communicable diseases among individuals with HIV infection by antiretroviral therapy status in Dar es Salaam, Tanzania. PloS one. 2020;15(7):e0235542.

98. Kazooba P, Kasamba I, Mayanja BN, Lutaakome J, Namakoola I, Salome T, et al. Cardiometabolic risk among HIV-POSITIVE Ugandan adults: prevalence, predictors and effect of long-term antiretroviral therapy. The Pan African medical journal. 2017;27:40.

99. Kilewo C, Natchu UC, Young A, Donnell D, Brown E, Read JS, et al. Hypertension in pregnancy among HIV-infected women in sub-Saharan Africa: prevalence and infant outcomes. African journal of reproductive health. 2009 Dec;13(4):25–36.

100. Kourtis AP, Bansil P, Kahn HS, Posner SF, Jamieson DJ. Diabetes trends in hospitalized HIV-infected persons in the United States, 1994-2004. Current HIV research. 2009 Sep;7(5):481–6.

101. Kuber A, Reuter A, Geldsetzer P, Chimbindi N, Moshabela M, Tanser F, et al. The effect of eligibility for antiretroviral therapy on body mass index and blood pressure in KwaZulu-Natal, South Africa. Sci Rep. 2021 Jul 19;11(1):14718.

102. Kwarisiima D, Balzer L, Heller D, Kotwani P, Chamie G, Clark T, et al. Population-Based Assessment of Hypertension Epidemiology and Risk Factors among HIV-Positive and General Populations in Rural Uganda. PloS one. 2016;11(5):e0156309.

103. Lubega G, Mayanja B, Lutaakome J, Abaasa A, Thomson R, Lindan C. Prevalence and factors associated with hypertension among people living with HIV/AIDS on antiretroviral therapy in Uganda. The Pan African medical journal. 2021;38:216.

104. Lukas K, Markos E, Belayneh F, Habte A. The magnitude of hypertension and associated factors among clients on highly active antiretroviral treatment in Southern Ethiopia, 2020: A hospital-based cross-sectional study. PLoS One. 2021;16(10):e0258576.

105. Maggi P, De Socio GV, Menzaghi B, Molteni C, Squillace N, Taramasso L, et al. Growing old with antiretroviral therapy or elderly people in antiretroviral therapy: two different profiles of comorbidity? BMC Infect Dis. 2022 Sep 23;22(1):745.

106. Magodoro IM, Okello S, Dungeni M, Castle AC, Mureyani S, Danaei G. Association between HIV and Prevalent Hypertension and Diabetes Mellitus in South Africa: Analysis of a Nationally Representative Cross-Sectional Survey. Int J Infect Dis. 2022 Aug;121:217–25.

107. Malaza A, Mossong J, Bärnighausen T, Newell ML. Hypertension and obesity in adults living in a high HIV prevalence rural area in South Africa. PloS one. 2012;7(10):e47761.

108. Malindisa E, Balandya E, Njelekela M, Kidenya BR, Francis F, Mmbaga BT, et al. Metabolic syndrome among people living with HIV on antiretroviral therapy in Mwanza, Tanzania. BMC Endocr Disord. 2023 Apr 21;23(1):88.

109. Manavalan P, Madut DB, Hertz JT, Thielman NM, Okeke NL, Mmbaga BT, et al. Hypertension burden and challenges across the hypertension treatment cascade among adults enrolled in HIV care in northern Tanzania. Journal of clinical hypertension (Greenwich, Conn). 2020 Aug;22(8):1518–22.

110. Manavalan P, Madut DB, Hertz JT, Thielman NM, Okeke NL, Mmbaga BT, et al. Hypertension among adults enrolled in HIV care in northern Tanzania: comorbidities, cardiovascular risk, and knowledge, attitudes and practices. Pan Afr Med J. 2022;41:285.

111. Masenga SK, Toloka P, Chiyenu K, Imasiku I, Mutengo H, Ulungu ON, et al. Type 2 diabetes mellitus prevalence and risk scores in treated PLWHIV: a cross-sectional preliminary study. BMC research notes. 2019 Mar;12(1):145.

112. Masyuko SJ, Page ST, Kinuthia J, Osoti AO, Polyak SJ, Otieno FC, et al. Metabolic syndrome and 10-year cardiovascular risk among HIV-positive and HIV-negative adults: A cross-sectional study. Medicine. 2020 Jul;99(27):e20845.

113. Mathabire Rücker SC, Tayea A, Bitilinyu-Bangoh J, Bermúdez-Aza EH, Salumu L, Quiles IA, et al. High rates of hypertension, diabetes, elevated low-density lipoprotein cholesterol, and cardiovascular disease risk factors in HIV-infected patients in Malawi. AIDS (London, England). 2018 Jan;32(2):253–60.

114. Mathebula RL, Maimela E, Ntuli NS. The prevalence of selected non-communicable disease risk factors among HIV patients on anti-retroviral therapy in Bushbuckridge sub-district, Mpumalanga province. BMC public health. 2020 Feb;20(1):247.

115. Mayer KH, Loo S, Crawford PM, Crane HM, Leo M, DenOuden P, et al. Excess Clinical Comorbidity Among HIV-Infected Patients Accessing Primary Care in US Community Health Centers. Public health reports (Washington, DC : 1974). 2018 Jan;133(1):109–18.

116. McGettigan P, Morales DR, Moreno-Martos D, Matin N. Changing co-morbidity and increasing deprivation among people living with HIV: UK population-based cross-sectional study. HIV Med. 2023 Mar;24(3):311–24.

117. Medina-Torne S, Ganesan A, Barahona I, Crum-Cianflone NF. Hypertension is common among HIV-infected persons, but not associated with HAART. Journal of the International Association of Physicians in AIDS Care (Chicago, Ill : 2002). 2012 Jan;11(1):20–5.

118. Melo ES, Antonini M, Costa CRB, Sorensen W, Gir E, Reis RK. Evaluation of cardiovascular risk factors in people living with HIV in São Paulo, Brazil. Journal of infection in developing countries. 2020 Jan;14(1):89–96.

119. Memiah P, Nkinda L, Majigo M, Opanga Y, Humwa F, Inzaule S, et al. Hypertension and Associated Inflammatory Markers Among HIV-Infected Patients in Tanzania. J Interferon Cytokine Res. 2021 Aug;41(8):291–301.

120. Mogaka JN, Sharma M, Temu T, Masyuko S, Kinuthia J, Osoti A, et al. Prevalence and factors associated with hypertension among adults with and without HIV in Western Kenya. PloS one. 2022;17(1):e0262400.

121. Mogaka JN, Lagat H, Otieno G, Macharia P, Wamuti B, Masyuko S, et al. Descriptive study: Feasibility of integrating hypertension screening into HIV assisted partner notification services model in Kenya. Medicine (Baltimore). 2023 Feb 22;102(8):e33067.

122. Muronya W, Sanga E, Talama G, Kumwenda JJ, van Oosterhout JJ. Cardiovascular risk factors in adult Malawians on long-term antiretroviral therapy. Transactions of the Royal Society of Tropical Medicine and Hygiene. 2011 Nov;105(11):644–9.

123. Musekwa R, Hamooya BM, Koethe JR, Nzala S, Masenga SK. Prevalence and correlates of hypertension in HIV-positive adults from the Livingstone Central Hospital, Zambia. The Pan African medical journal. 2021;39:237.

124. Mwakyandile TM, Shayo GA, Sasi PG, Mugusi FM, Barabona G, Ueno T, et al. Hypertension and traditional risk factors for cardiovascular diseases among treatment naïve HIV- infected adults initiating antiretroviral therapy in Urban Tanzania. BMC Cardiovasc Disord. 2023 Jun 20;23(1):309.

125. Myerson M, Poltavskiy E, Armstrong EJ, Kim S, Sharp V, Bang H. Prevalence, treatment, and control of dyslipidemia and hypertension in 4278 HIV outpatients. Journal of acquired immune deficiency syndromes (1999). 2014 Aug;66(4):370–7.

126. Nartey ET, Tetteh RA, Anto F, Sarfo B, Kudzi W, Adanu RM. Hypertension and associated factors among patients attending HIV clinic at Korle-Bu Teaching Hospital. Ghana Med J. 2023 Jan;57(1):19–27.

127. Ngu RC, Choukem SP, Dimala CA, Ngu JN, Monekosso GL. Prevalence and determinants of selected cardio-metabolic risk factors among people living with HIV/AIDS and receiving care in the South West Regional Hospitals of Cameroon: a cross-sectional study. BMC research notes. 2018 May;11(1):305.

128. Niwaha AJ, Wosu AC, Kayongo A, Batte C, Siddharthan T, Kalyesubula R, et al. Association between Blood Pressure and HIV Status in Rural Uganda: Results of Cross-Sectional Analysis. Global heart. 2021 Feb;16(1):12.

129. Niwaha AJ, Wosu AC, Namugenyi C, Kayongo A, Nyirenda MJ, Siddharthan T, et al. 24-hour ambulatory blood pressure monitoring and hypertension related risk among HIV-positive and HIV-negative individuals: cross sectional study findings from rural Uganda. J Hum Hypertens. 2022 Feb;36(2):144–52.

130. Njoroge A, Augusto O, Page ST, Kigondu C, Oluka M, Puttkammer N, et al. Increased risk of prediabetes among virally suppressed adults with HIV in Central Kenya detected using glycated haemoglobin and fasting blood glucose. Endocrinol Diabetes Metab. 2021 Oct;4(4):e00292.

131. Ogunmola OJ, Oladosu OY, Olamoyegun AM. Association of hypertension and obesity with HIV and antiretroviral therapy in a rural tertiary health center in Nigeria: a cross-sectional cohort study. Vascular health and risk management. 2014;10:129–37.

132. Ojong E, Iya B, Djeufouata J, Ndeh F, Nsonwu A, Njongang V, et al. Metabolic syndrome and its components among HIV/AIDS patients on Antiretroviral Therapy and ART-Naïve Patients at the University of Calabar Teaching Hospital, Calabar, Nigeria. Afr Health Sci. 2022 Mar;22(1):410–7.

133. Ottaru TA, Kwesigabo GP, Butt Z, Rivera AS, Chillo P, Siril H, et al. Ideal Cardiovascular Health: Distribution, Determinants and Relationship with Health Status among People Living with HIV in Urban Tanzania. Glob Heart. 2022;17(1):74.

134. Oyawa I, Adhiambo M, Wesonga B, Wanzala M, Adungo F, Makwaga O, et al. Burden of hypertension and associated factors among HIV-positive adults in Busia County, Kenya. Pan Afr Med J. 2022;43:143.

135. Peck RN, Shedafa R, Kalluvya S, Downs JA, Todd J, Suthanthiran M, et al. Hypertension, kidney disease, HIV and antiretroviral therapy among Tanzanian adults: a cross-sectional study. BMC medicine. 2014 Jul;12:125.

136. Pérez-Chaparro CGA, Schuch FB, Zech P, Kangas M, Rapp MA, Heissel A. Recreational Exercising and Self-Reported Cardiometabolic Diseases in German People Living with HIV: A Cross-Sectional Study. Int J Environ Res Public Health. 2021 Nov 4;18(21).

137. Pierre S, Seo G, Rivera VR, Walsh KF, Victor JJ, Charles B, et al. Prevalence of hypertension and cardiovascular risk factors among long-term AIDS survivors: A report from the field. Journal of clinical hypertension (Greenwich, Conn). 2019 Oct;21(10):1558–66.

138. Puhr R, Petoumenos K, Huang R, Templeton DJ, Woolley I, Bloch M, et al. Cardiovascular disease and diabetes in HIV-positive and HIV-negative gay and bisexual men over the age of 55 years in Australia: insights from the Australian Positive & Peers Longevity Evaluation Study. HIV medicine. 2019 Feb;20(2):121–30.

139. Rajagopaul A, Naidoo M. Prevalence of diabetes mellitus and hypertension amongst the HIV-positive population at a district hospital in eThekwini, South Africa. Afr J Prim Health Care Fam Med. 2021 Sep 29;13(1):e1–6.

140. Rhee JY, Bahtila TD, Palmer D, Tih PM, Aberg JA, LeRoith D, et al. Prediabetes and diabetes among HIV-infected adults in Cameroon. Diabetes/metabolism research and reviews. 2016 Sep;32(6):544–9.

141. Russell E, Albert A, Côté H, Hsieh A, Nesbitt A, Campbell AR, et al. Rate of dyslipidemia higher among women living with HIV: A comparison of metabolic and cardiovascular health in a cohort to study aging in HIV. HIV medicine. 2020 Aug;21(7):418–28.

142. Sander LD, Newell K, Ssebbowa P, Serwadda D, Quinn TC, Gray RH, et al. Hypertension, cardiovascular risk factors and antihypertensive medication utilisation among HIV-infected individuals in Rakai, Uganda. Tropical medicine & international health : TM & IH. 2015 Mar;20(3):391–6.

143. Seang K, Javanbakht M, Lee SJ, Brookmeyer R, Pheng P, Chea P, et al. Differences in prevalence and risk factors of non-communicable diseases between young people living with HIV (YLWH) and young general population in Cambodia. PLoS One. 2022;17(6):e0269989.

144. Serrão R, Piñero C, Velez J, Coutinho D, Maltez F, Lino S, et al. Non-AIDS-related comorbidities in people living with HIV-1 aged 50 years and older: The AGING POSITIVE study. International journal of infectious diseases : IJID : official publication of the International Society for Infectious Diseases. 2019 Feb;79:94–100.

145. Shidhaye P, Ghate M, Gurav S, Gupte MD, Panda S. Metabolic syndrome in people living with antiretroviral therapy: A cross-sectional investigation from Pune, India. Indian J Public Health. 2023 Mar;67(1):84–91.

146. Steiniche D, Jespersen S, Erikstrup C, Krarup H, Handberg A, Østergaard L, et al. Diabetes mellitus and impaired fasting glucose in ART-naïve patients with HIV-1, HIV-2 and HIV-1/2 dual infection in Guinea-Bissau: a cross-sectional study. Transactions of the Royal Society of Tropical Medicine and Hygiene. 2016 Apr;110(4):219–27.

147. Tadesse WT, Adankie BT, Shibeshi W, Amogne W, Aklillu E, Engidawork E. Prevalence and predictors of glucose metabolism disorders among People Living with HIV on combination antiretroviral therapy. PLoS One. 2022;17(1):e0262604.

148. Trifirò S, Cavallin F, Mangi S, Mhaluka L, Maffoni S, Taddei S, et al. Hypertension in people living with HIV on combined antiretroviral therapy in rural Tanzania. Afr Health Sci. 2023 Mar;23(1):129–36.

149. Tsuro U, Oladimeji KE, Pulido-Estrada GA, Apalata TR. Risk Factors Attributable to Hypertension among HIV-Infected Patients on Antiretroviral Therapy in Selected Rural Districts of the Eastern Cape Province, South Africa. Int J Environ Res Public Health. 2022 Sep 6;19(18).

150. van Zoest RA, Wit FW, Kooij KW, van der Valk M, Schouten J, Kootstra NA, et al. Higher Prevalence of Hypertension in HIV-1-Infected Patients on Combination Antiretroviral Therapy Is Associated With Changes in Body Composition and Prior Stavudine Exposure. Clinical infectious diseases : an official publication of the Infectious Diseases Society of America. 2016 Jul;63(2):205–13.

151. Wallace DE, Horberg MA, Benator DA, Greenberg AE, Castel AD, Monroe AK, et al. Diabetes mellitus control in a large cohort of people with HIV in care-Washington, D.C. AIDS Care. 2021 Nov;33(11):1464–74.

152. Willig AL, Westfall AO, Overton ET, Mugavero MJ, Burkholder GA, Kim D, et al. Obesity is associated with race/sex disparities in diabetes and hypertension prevalence, but not cardiovascular disease, among HIV-infected adults. AIDS research and human retroviruses. 2015 Sep;31(9):898–904.

153. Wu PY, Chen MY, Hsieh SM, Sun HY, Tsai MS, Lee KY, et al. Comorbidities among the HIV-infected patients aged 40 years or older in Taiwan. PloS one. 2014;9(8):e104945.

154. Ximenes RA, Lacerda HR, Miranda-Filho DB, Albuquerque Mde F, Montarroyos UR, Turchi MD, et al. Comparison between potential risk factors for cardiovascular disease in people living with HIV/AIDS in areas of Brazil. Journal of infection in developing countries. 2015 Sep;9(9):988–96.
